# Extracellular shuttling miR‐21 contributes to esophageal cancers and human umbilical vein endothelial cell communication in the tumor microenvironment and promotes tumor angiogenesis by targeting phosphatase and tensinhomolog

**DOI:** 10.1111/1759-7714.15103

**Published:** 2023-09-19

**Authors:** Shanbo Zheng, Juan Liao, Mingjun Sun, Ran Liu, Junjie Lv

**Affiliations:** ^1^ Department of Thoracic Surgery and State Key Laboratory of Genetic Engineering Fudan University Shanghai Cancer Center Shanghai People's Republic of China; ^2^ Institute of Thoracic Oncology Fudan University Shanghai People's Republic of China; ^3^ Department of Oncology, Shanghai Medical College Fudan University Shanghai People's Republic of China; ^4^ Key Laboratory of Environmental Medicine Engineering, Ministry of Education, School of Public Health Southeast University Nanjing People's Republic of China; ^5^ Department of Science and Education, Affiliated Hangzhou First People's Hospital Zhejiang University School of Medicine Hangzhou People's Republic of China

**Keywords:** angiogenesis, ESCC, exo‐miR‐21, PTEN/Akt, scRNA‐seq

## Abstract

**Background:**

Cell‐cell communication by carcinoma‐derived exosomes can influence the tumor microenvironment (TME) and regulate cancer progression. Based on the overexpression of microRNA‐21‐5p (miR‐21) in plasma from patients diagnosed with esophageal squamous cell carcinoma (ESCC) and exosomes from ESCC cell lines identified earlier, this study aimed to explore the influence of exosomal miR‐21 within the TME.

**Method:**

ScRNA‐Seq and Bulk RNA‐Seq were integrated to elucidate the communication between cancer and endothelial cells. The functionality and mechanisms by which exo‐miR‐21 derived from carcinoma regulate endothelial cell‐mediated angiogenesis were assessed using a cocultivation model of EC9706 cells and recipient human umbilical vein endothelial cells (HUVECs), through blood vessel formation experiments, luciferase reporter assays, RT‐qPCR, and western blot analysis.

**Result:**

A total of 3842 endothelial cells were extracted from the scRNA‐seq data of ESCC samples and reclustered into five cell subtype. Cell‐cell communication analysis revealed cancer cells presented a strong interaction with angiogenesis‐like endothelial cells in secreted signaling. MiR‐21 was unregulated in ESCC and the carcinoma‐derived exo‐miR‐21 was significantly raised in HUVECs. The exo‐miR‐21 promoted the proliferation and migration of HUVECs while also enhancing, closed mesh count, and junction number in HUVECs. Mechanistically, dual‐luciferase reporter assay revealed that PTEN was the target of miR‐21. Meanwhile, p‐Akt was significantly increased and suppressed by inhibition of miR‐21 and PI3K inhibitor LY294002.

**Conclusion:**

Exo‐miR‐21‐mediated communication between endothelial and cancer cells plays a pivotal role in promoting the angiogenesis of ESCC. Therefore, controlling exo‐miR‐21 could serve as a novel therapeutic strategy for ESCC by targeting angiogenesis.

## Graphical abstract

Schematic representation of carcinoma‐derived exosomal microRNA‐21‐5p (miR‐21) promote angiogenesis via regulating the PTEN/Akt signaling pathway in endothelial cells.

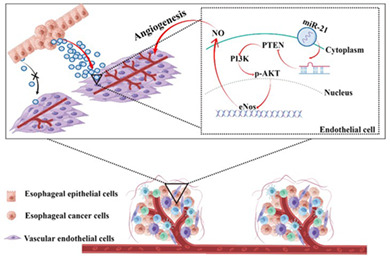

## INTRODUCTION

Esophageal carcinoma (EC) is the sixth leading cause of cancer‐related deaths in mortality worldwide.^1^ Among the subtypes of EC, esophageal cell squamous carcinoma (ESCC) mainly occurs in Asia and east Africa, and is ascribed to multiple risk factors, genes, and stages.^2^, ^3^ Despite advances in surgical, radiotherapeutic, and chemotherapeutic approaches, the overall five‐year survival for ESCC patients remains below 15% due to late‐stage diagnosis.^4^ Therefore, searching for biomarkers to effectively control tumor metastasis may prolong the survival time of patients.

Tumor metastasis is affected by a variety of factors, yet emerging evidence has shown new vessel formation (angiogenesis) contributes greatly to it.^5^, ^6^, ^7^ Angiogenesis is known as a hallmark of tumors and key promoter of tumor growth and recurrence and the resultant tumor microenvironment (TME) contribute to diminished efficacy of chemotherapy, radiotherapy, and immunotherapy while facilitating cancer progression.^6^ Our previous meta‐analysis also revealed a significant correlation between high microvessel density and poor clinicopathological criteria as well as reduced survival rates in patients with ESCC.^8^ Therefore, further exploration into the molecular mechanism of tumor angiogenesis in TME is needed and normalization of the tumor vasculature may offer a novel therapeutic strategy for ESCC.

Exosomes are small extracellular vesicles, ranging in size from 30 to 150 nm, that are produced by various cells. Exosomes contain a variety of biologically active molecules, such as microRNAs (miRNAs), messenger RNAs (mRNA), DNA fragments and proteins.^9^, ^10^ The transferred molecules can effectively modulate cellular functions and impact receptor cell processes, particularly enhancing intercellular interactions within the TME.^10^, ^11^ It is worth mentioning that tumor cells secrete millions of exosomes, which are disseminated throughout the TME. A previous study by Whiteside suggested that patients with cancer, especially those with advanced or metastatic disease, have greatly increased numbers of exosomes in the plasma compared to healthy blood donors.^12^ The majority of studies have proved that exosomal miRNAs play a part in the development, metastasis and drug resistance of cancer.^13^, ^14^ As an important component of the TME, exosomes play a critical role in angiogenesis by transporting vascular endothelial growth factor (VEGF), matrix metalloproteinases (MMP), miRNAs and many other proangiogenic biomolecules.^15^ Existing research showed that miRNAs promote angiogenesis and neoplastic processes in various cancers including breast cancer, colorectal cancer papillary thyroid carcinoma and so on.^16^, ^17^, ^18^

Our previous study found that the occurrence of ESCC was significantly associated with plasma microRNA‐21‐5p (miR‐21), and demonstrated that a close correlation exists between exosome‐shuttling miR‐21 and EC recurrence and distant metastasis.^19^ Existing studies proved that miR‐21 has multiple functions in human cancers, including promoting cell proliferation, migration, invasion, metastasis, through regulating a series of pathways including the PI3K/Akt/mTOR^20^ and RAS/MEK/ERK pathways.^21^ Our previous next‐generation sequencing data showed that miR‐21 was abundant in both ESCC cells and their corresponding exosomes.^22^ However, the mechanism of exosomal miR‐21 leading to angiogenesis in human umbilical vein endothelial cells (HUVECs) is not clear enough. The aim of this study was to elucidate the mechanism of exosomal miR‐21 from ESCC cells promoting angiogenesis in HUVECs, which might serve as a novel therapeutic target for ESCC.

## METHODS

### Single‐cell RNA‐seq data acquisition and processing

The scRNA‐seq data of ESCC samples (GSE188900) were collected from the Gene Expression Omnibus (GEO) database from the official website (https://www.ncbi.nlm.nih.gov/gds). The “Seurat”, “LogNormalize” and “VST” were used in R and RStudio software to perform the integration of the scRNA‐seq data, normalized and identified the hypervariable genes, respectively. The cells with 200–5000 gene features were included. The“FindNeighbors”and “FindClusters”function were used to evaluate the number of cell clusters. Under the thresholds that *p* < 0.05 and |log2FoldChange (FC)| > 1, the “FindAllMarkers” was used to identify the marker genes. Finally, the results were visualized by the t‐SNE and UMAP methods.

### Extraction and identification of endothelial cells and epithelial cells

The automated annotation “SingleR” package was used to further extract and identify the endothelial and epithelial cells, and was then modified according to the known cell markers of previous studies. Specifically, the following marker genes were used for extraction and identification of cell types, endothelial cells: VWF, CDH5, RAMP2 and CLDN5; epithelial cells: SPRR3, SFN, keratin genes (KRT5, KRT7, KRT8, KRT14, KRT17) and EPCAM. In addition, subclustering was performed for endothelial cells for more in‐depth investigation and better annotation of clusters according to the more specific markers.

### Pseudotime trajectory analysis

The R package “monocle2” was used to infer the trajectory of epithelial cells, fibroblasts, T cells and macrophages. The “NewCellDataSet” function was performed to construct the “monocle” object, and the differentially expressed genes (DEGs) were screened by “differentialGeneTest” function in Seurat. Then, the “DDRTree” method and “ggsave” function were applied to dimension reduction and visualization of results, respectively.

### Gene set variation analysis (GSVA)

The R package “GSVA” was used based on the hallmark genes that were defined as the top 50 genes with the largest fold change (FC) in endothelial cell subclustering. The up‐ and downregulated functional pathways were visualized by heatmap.

### Cell‐cell communication analysis

Cell‐Cell communication was identified by the “CellPhoneDB”. The ligand‐receptor pairs were retrieved from CellPhoneDB database (https://www.cellphonedb.org/). Then, the “CellChat” object was created using the “createCellChat” function, and the cellular communication network was inferred based on the “CellChat”. Finally, the result was visualized by “ggplot2”.

### Cell culture and cell transfection

The human EC cell line EC9706 (National Laboratory of Molecular Oncology, Beijing, China) was cultured in RPMI‐1640 (Gibco) medium. HUVECs (Shanghai Institute of Biochemistry and Cell Biology, CAS) were cultured in F12K medium (Gibco). Both of the basal culture media above were supplemented with 10% fetal bovine serum (FBS; Gibco) and 1% penicillin and streptomycin in a 5% CO_2_ incubator at 37°C. The exosome‐free FBS was obtained by centrifugation at 200, 000 × *g* for 6 h at 4°C to removal the bovine‐derived exosomes. EC9706 cells and HUVECs were seeded into six‐well plates and transfected using Lipofectamine RNAiMAX (Invitrogen) and Opti‐MEM (Gibco), following the manufacturers’ instructions. An inverted fluorescence microscope was used to detect the transfer efficiency of miR‐21 mimics, inhibitors, and normal control (NC). The miR‐21 expression level was detected by real‐time quantitative polymerase chain reaction (RT‐qPCR).

### Exosome isolation and identification

Exosomes were isolated from cell culture medium by differential centrifugation according to a previously described procedure.^23^ Briefly, 100 mL of the cellular supernatant was centrifuged at 300 × *g* for 10 min, 1200 × *g* for 20 min, and 10 000 × *g* for 30 min at 4°C to remove the cell debris and collect the supernatant. Then, the collected supernatant was centrifuged at 200 000 × *g* for 2 h and purified by centrifugation at 100 000 × *g* for 1 h, and then stored at −80°C after resuspension in phosphate buffered saline (PBS). Transmission electron microscopy (TEM) were used for the morphological characteristic identification and size distribution.

### Exosomal miR‐21 internalization into HUVECs cells

EC9706 cells transfected with Cy3‐miR‐21 mimics were seeded into the upper chambers of six‐well transwell inserts at a 1 × 10^5^ cells/well density. Then, the HUVECs were preseeded in the lower chambers at the same cell density. After 24 h, the fluorescence microscopy used to assay the internalization level of exosomal miR‐21. The miR‐21 expression level in HUVECs was detected by RT‐qPCR. Cocultivation of donor EC9706 cells and recipient HUVECs were performed in 12‐well transwell inserts (Corning). HUVECs were preseeded in the lower chambers at a 1 × 10^5^ cells/well density. Then, the EC9706 cells were transfected with miR‐21 mimics or the NC and seeded into 0.4 μM transwell inserts the following day.

### RNA extraction and RT‐qPCR

Total RNA was extracted from the cultured cells and exosomes using TRIzol reagent (Invitrogen) and mirVana miRNA isolation kit (Ambion) according to the protocols of manufacturer, respectively. RNA concentration was measured using a NanoDrop spectrophotometer (NanoDrop ND‐1000). Then, the expression of PTEN and miR‐21 were quantified using the SYBR Green mastermix (Vazyme) in a StepOne Plus System (Applied Biosystems). Transcription levels were normalized to the internal reference level (β‐actin or U6) and presented as fold changes using the 2^−ΔΔCT^ method. The sequences of target genes are presented in Table S1.

### Relative expression of miR‐21 in ESCC and the miRNA target gene

The website of https://www.xiantao.love/products was used to analyze the expression of miR‐21 in ESCC and draw the receiver operating curve (ROC). TargetMiner (https://www.isical.ac.in/~bioinfo_miu/targetminer20.htm), miRwalk (http://zmf.umm.uni-heidelberg.de/), RNA22 (https://cm.jefferson.edu/rna22/Interactive) were used to predict the miRNA target and binding site, and DIANA (http://diana.imis.athena-innovation.gr/DianaTools/index.php) was used to analyze the results. MiR‐PTEN plasmid was obtained from RiboBio. HUVECs in the miR‐21 mimics or NC group were transfected into 96‐well plates (1 × 10^4^ cells/well) with pmiR‐Report‐WT‐PTEN by Lipofectamine RNAiMAX. Dual luciferase assay kit (Promega) was used to analyze the miRNA and target gene according to guidelines of manufacturer.

### Luciferase reporter assay

The reporter plasmid pmiR‐PTEN was designed by RiboBio. EC9706 cells in the miR‐21 mimics or negative control group were transfected into 96‐well plates (1 × 10^4^ cells/well) with pmiR‐Report‐WT‐PTEN by Lipofectamine RNAiMAX. The renilla luciferase was used as an internal control. At 48 h after transfection, cells were analyzed using a dual luciferase assay kit (Promega).

### Vascular ring formation by HUVECs in vitro angiogenesis assay

An in vitro angiogenesis assay was performed as previously described.^24^, ^25^ Briefly, 30 μL Matrigel (BD Biosciences) was added to 96‐well plate, and then polymerized at 37°C for 30 min. Next, the pretreated HUVECs were resuspended in FBS‐free RPMI‐1640 medium and transferred to each well at a concentration of 4 × 10^4^ cells per well. After 12 h, the cells were examined under an Olympus FSX100 light microscope to assess the formation of capillary‐like structures. The total tubule length, number of closed meshes and branch points of the formed tubes, which represent the degree of angiogenesis in vitro, were scanned and quantified in at least 10 low‐power fields (40× magnification).

### Cell proliferation assay

The proliferative ability of HUVECs after different transfections or different exosomes treated was determined by Cell‐Light 5‐ethynyl‐2′‐deoxyuridine (EdU) Apollo in vitro image kit (RiboBio). After pretreatment as described above, HUVECs were incubated in 50 μM EdU for 2 h, and were then fixed and stained following the appropriate manufacturer's instructions.

### Cell migration assay

The migratory capacity of HUVECs was performed using a transwell insert that contains a polycarbonate filter with 8 μM pore size (Corning). The pretreated HUVECs cells (5 × 10^3^/well) suspended in 150 μL serum‐free RPMI‐1640 were added to the 24‐well upper chamber, and 600 μL RPMI‐1640 that contained 10% FBS was added to the bottom wells. Cells were incubated at 37°C for 24 h to allow cell migration through the membrane. Ethanol was used to fix migrated cells and then stained with crystal violet. The number of invaded cells was counted under an Olympus FSX100 light microscope (Olympus). To minimize the bias, cells in 10 randomly selected fields at a 200× magnification were counted to calculate the average cell number.

### Western blot analysis

Cells were lysed in radioimmunoprecipitation assay (RIPA) buffer with protease inhibitor. After quantification by BCA kit (Beyotime Biotechnology), the denatured protein was separated on SDS‐PAGE gels and transferred to polyvinylidene fluoride (PVDF) membranes (Millipore). The immunoblots were blocked with 5% fat‐free milk and incubated at 4°C overnight with primary antibodies anti‐CD63 (1:1200, Abclone), anti‐PTEN (1:1000, Cell Signaling Technology), anti‐β‐actin (1:1000, Wuhan Boster Biological Technology, China), anti‐Akt and anti‐p‐Akt (Ser473) (1:1000, Cell Signaling Technology). After incubation with the secondary antibody, the bands were detected by enhanced chemiluminescence kit on in the Gel‐Pro Analyzer software.

### Statistical analysis

All values are described as mean ± standard deviation (SD) and representative of at least three independent experiments. Student's *t*‐test or Wilcoxon rank sum test were used for comparison of the continuous variables according to the data distribution. Spearman correlation test was applied to examine the correlations. Statistical analyses were performed with SPSS 16.0 software (SPSS) and Graphpad Prism 7.0 (Graphpad Software). The differences were considered statistically significant at *p* < 0.05.

## RESULTS

### Heterogeneity of endothelial cells in ESCC microenvironment

**FIGURE 1:**
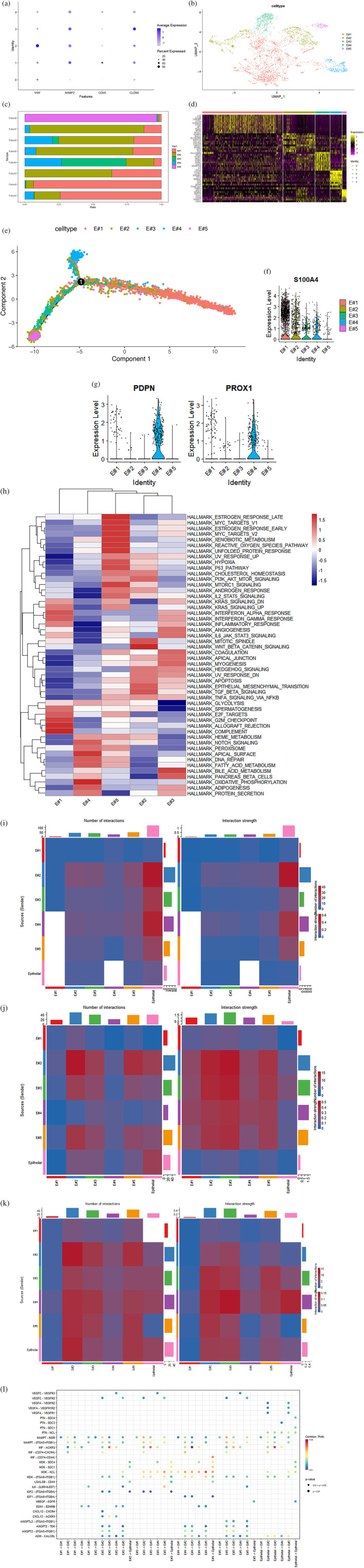
Cell‐cell communications between endothelial and esophageal cancer cells. (a) The expression distribution of fibroblast marker genes VWF, RAMP2, CDH5 andCLDN5 in five clusters. (b) The UMAP algorithm divided the 3842 endothelial cells into five subsets. (c) The distribution of all five endothelial cell subpopulations in each sample. (d) Heatmap showing the expression of marker genes in each state of endothelial cell subpopulations. (e) Trajectory analysis result of five endothelial cell types. Cell types were assigned by different colors and arranged by pseudo‐time. (f, g) The expression of S100A4 (f), PDPN ([f]‐left) and PROX1 ([f]‐right) were significantly enriched in fibroblast base on scRNA_seq data. (h) Heatmap showing the GSVA analysis of regulatory pathways in all five endothelial cell subsets. (i–k) Heatmap showing the interaction intensity between endothelial cells and cancer cells ([i]:ECM‐receptor; [j]:cell–cell contact; [k]:secreted signaling). (l) Bubble plot showing the selected ligand‐receptor interactions between cancer cells and each endothelial cell subsets.

A total of 3842 endothelial cells from seven tumor samples and one paracancerous tissue were obtained from the GEO database, based on the marker genes VWF, CDH5, RAMP2 and CLDN5 (Figure 1a),^26^, ^27^ of which 3724 and 118 cells were derived from cancer tissues and paracancerous tissue, respectively. Additionally, 21 273 genes were included for identify highly variable genes, and the top 2000 highly variable genes (G0S2, CCL21, SPP1, NTS, MMP1 and so on) were shown in Figure S1a. After the PCA and t‐SNE dimensionality reduction (Figures S2 and S3), five subsets of endothelial cells were identified by the “FindCluster” function in “resolution = 0.1”, including E#1, E#2, E#3, E#4 and E#5 (Figure 1b). Significantly, the P6‐n, a paracancerous tissue, was largely represented by E#5 subsets, whereas tumor samples mostly contained E#1‐E#4 subsets (Figure 1c). Further，the heatmap showed that highly expressed genes in different subsets, such as HLA‐DRB5 in E#3, CCL21 in E#4 and KRT4 in E#5，indicating that their distinct transcriptional programs and characteristic functions (Figure 1d). Importantly, pseudotime trajectory analysis indicated that starting from normal endothelial cells E#5, E#2 and E#3 spread along the axis scoring an excessive state, followed by differentiation to E#4 at one end and to E#1 at the other end (Figure 1e). Subsequently, the E#4 was defined as lymphatic endothelial (LEC) cells by the marker genes (LEC) (e.g., PDPN and PROX1), and E#1 was defined as carcinoma‐associated fibroblasts derived endothelial cells (CAFsEc) (e.g., S100A4) (Figure 1f,g). However, the GSVA functional analysis revealed E#2 and E#3 have a unique functional role, such as epithelial‐mesenchymal transition and angiogenesis (Figure 1h). Therefore, the E#2 and E#3 were defined as endothelial‐to‐mesenchymal transition (EndMT) and angiogenesis‐like endothelial cells (AEC). Collectively, these results revealed high degrees of heterogeneity in endothelial cells.

### Cell‐cell communications between endothelial and esophageal cancer cells

New angiogenesis was an important pathological process in tumorigenesis and progression, and it provides the oxygen and nutrients required for tumorigenesis. Further, pseudotime trajectory analysis indicated that AEC were in the transitional stage between normal endothelial cells and LEC, and CAF, EC, which may play a decisive role in the process of tumor progression, but the evolutionary mechanism and the communication relationship between cancer cells are still not clear. Here, we performed the “CellPhoneDB” to assess the crosstalk between various types of cells, including 61.8% secreted signaling, 21.7% ECM‐receptor and 16.5% cell‐cell contact (Figure S1c). In ECM‐receptor, cancer cells shown a strong interaction with EndMT (Figure 1i); In cell‐cell contact, cancer cells shown a strong interaction with EndMT and LEC (Figure 1j); Importantly, AEC presented a strong interaction with cancer cells in secreted signaling (Figure 1k,l and Figure S1b). In summary, cancer cells and AEC cells have significant interactions in the early secretory signaling interaction pathway, but the exact mode of interaction and mechanism are not yet clear.

### Exosome transported miR‐21 from EC9706 into HUVECs

**FIGURE 2:**
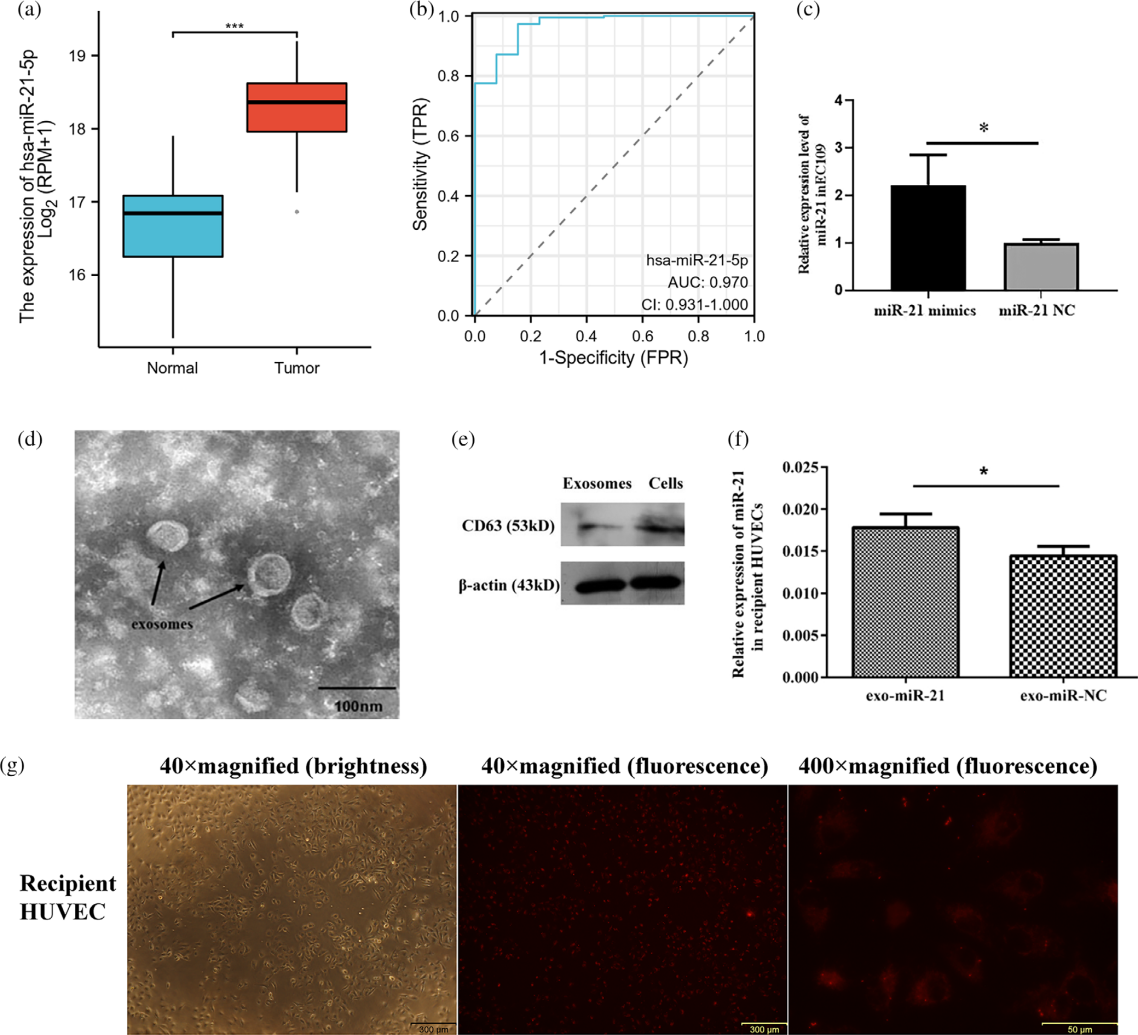
Exosomes transport miR‐21 from EC9706 into human umbilical vein endothelial cells (HUVECs). (a) The relative expression of miR‐21 in the tumor and Normal tissue. (b) The AUC of miR‐21. (c) The transfection efficiency of miR‐21 in EC9706. (d) Morphology of exosomes from EC9706 under electron microscope. (e) Expression detection of one of exosome marker proteins CD63. (f) The relative expression of miR‐21 in HUVECs after coculture with EC9706 cells. (g) miR‐21 transferred to HUVECs via EC9706 cell‐derived exosomes. *p < 0.05, ***p < 0.001.

The miR‐21 was upregulated in tumor tissue compared with normal tissue (Figure 2a), and the ROC‐AUCs of miR‐21 was 0.906 (Figure 2b). These results indicated that miR‐21 might exert a pivotal influence on the tumor progression. In order to explore the function of exosomes miR‐21 in tumors, we constructed overexpression miR‐21 EC9706 by transfection with Cy3‐labeled miR‐21 mimics. After 24 h, the transfection efficiency was evaluated by RT‐qPCR, and shown that miR‐21 expression level was significantly increased in the miR‐21mimics group with an average fold change of 3014 (*p* < 0.05) compared with NC mimics group (Figure 2c). We extracted exosomes from EC9706 cells and identified by TEM, and the results shown that exosomes exhibited a round‐shaped morphology and the diameter of particles was approximately concentrated at 55 nm (Figure 2d). Further, exosome markers CD63 was positive in the supernatant from EC9706 cells (Figure 2e). Subsequently, to determine whether exosome‐delivered miR‐21 can be shuttled into HUVECs, we constructed a cocultivation of HUVECs with EC9706 cells. After cocultivation with EC9706 cells transfected with miR‐21 mimics and HUVECs for 24 h, the miR‐21 content in HUVECs was rapidly expanding (Figure 2g), miR‐21 expression levels were obviously upregulated in HUVECs which cocultured with EC9706 cells transfected with miR‐21 mimics, with an average fold change of 1.23 compared with the control group (Figure 2f). These results demonstrated the secretion of miR‐21 from EC9706 cells and its delivery into HUVECs cells via exosomes.

### Exosome‐shuttling miR‐21 promoted HUVECs angiogenesis activity

**FIGURE 3:**
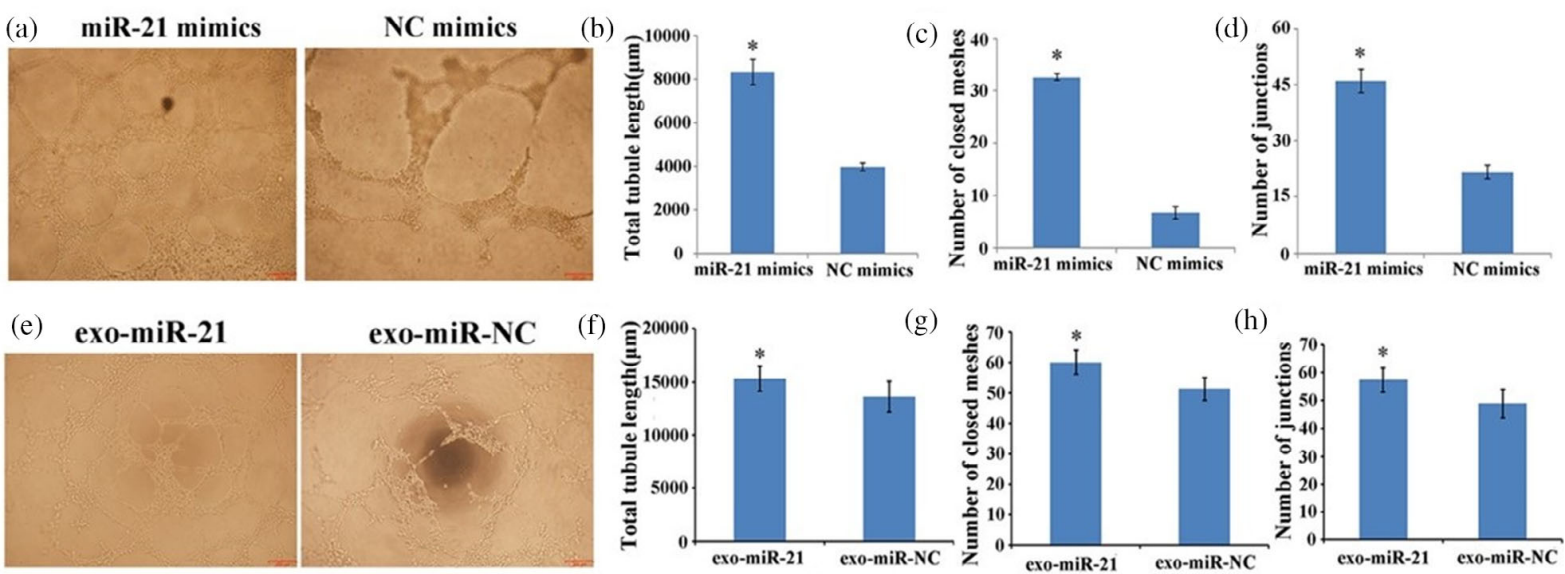
MiR‐21 and EC9706‐Exo delivered miR‐21 promotes angiogenesis. (a) Human umbilical vein endothelial cell (HUVEC) tube formation after treatment with or without a miR‐21 mimic. Scale bar: 200 μm. (b–d) Quantitative evaluation of tube length, number of closed meshes and number of junctions. (e) HUVEC tube formation after coculture with EC9706 cells transfected with miR‐21 mimics. Scale bar: 200 μm. (f–h) Quantitative evaluation of tube length, number of closed meshes and number of junctions in HUVECs after coculture with EC9706 cells transfected with miR‐21 mimics. *p < 0.05.

Tube formation assay was conducted to further evaluate the effects of exosome‐shuttling miR‐21 on angiogenesis in vitro. HUVECs were treated with miR‐21 mimics and NC mimics for 48 h, the tube length (Figure 3b), the number of closed meshes (Figure 3c) and the number of junctions (Figure 3d) in HUVECs (Figure 3a) were significantly increased in the miR‐21mimics treated group with an average fold change of 2.11, 4.83, 2.13 (*p* < 0.05) compared with the NC group. After coculture with EC9706 cells transfected with miR‐21 mimics for 24 h, the tube length (Figure 3f), number of closed meshes (Figure 3g) and number of junctions (Figure 3h) in HUVECs (Figure 3e) were also increased compared with the miR‐NC mimics group (*p* < 0.05). The results showed that exosome‐shuttling miR‐21 promoted the angiogenesis of HUVECs.

### Exosome‐shuttling miR‐21 promoted the proliferation and migration of HUVECs

**FIGURE 4:**
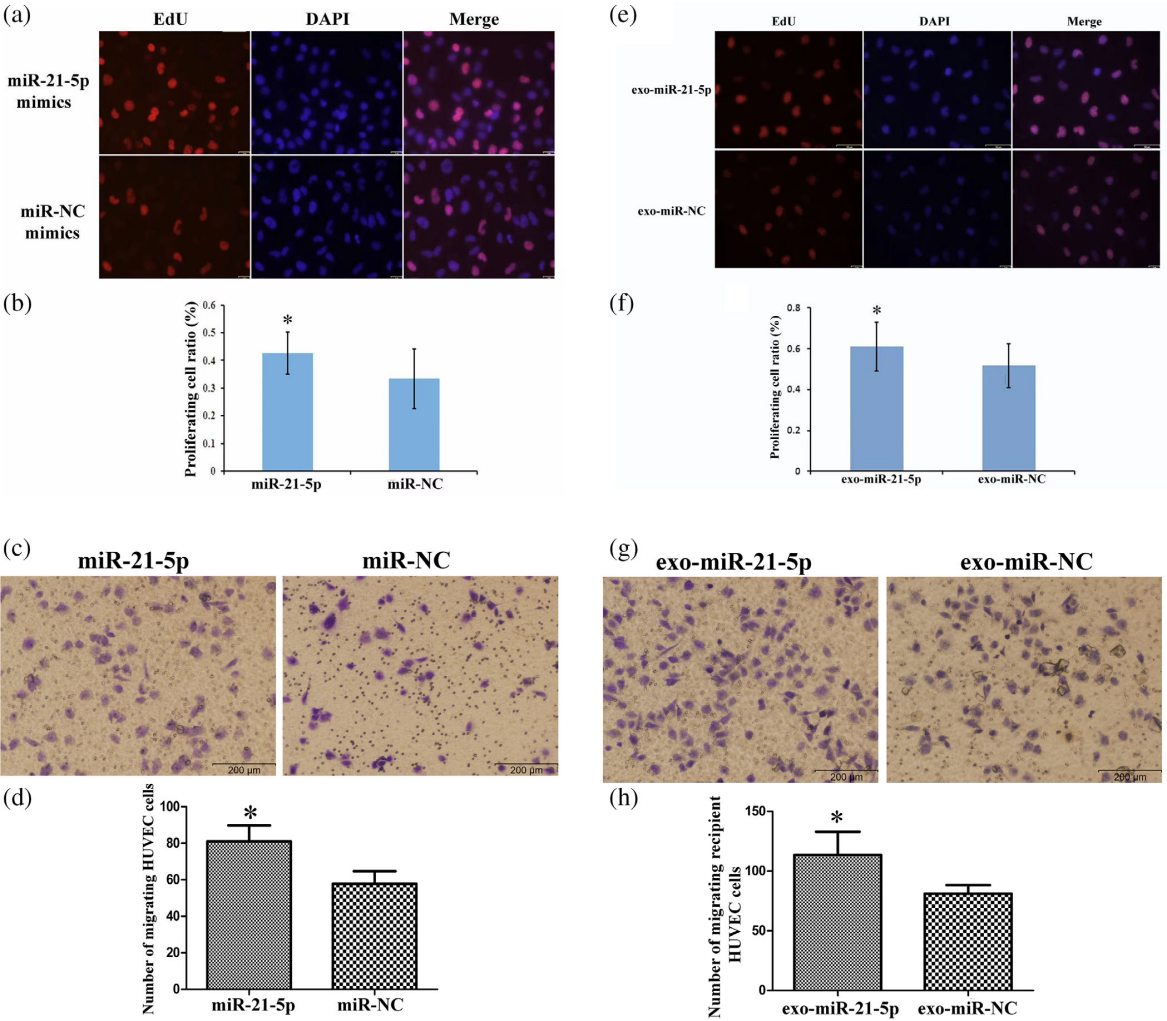
MiR‐21 and EC9706 delivered exo‐miR‐21 promotes endothelial cell growth and migration. (a, b) 5‐ethynyl‐2′‐deoxyuridine (EdU) assays revealed that miR‐21 mimics promoted the proliferation of human umbilical vein endothelial cells (HUVECs). (c, d) Transwell assays demonstrated that miR‐21 mimics enhanced the migration ability of HUVECs. (e, f) EdU assays revealed that EC9706‐Exo delivered miR‐21 promoted the proliferation of HUVECs. (g, h) Transwell assays demonstrated that EC9706‐Exo delivered miR‐21 enhanced the migration ability of HUVECs. *p < 0.05.

EdU proliferation and migration assays were used to confirm the impact of exosome‐shuttling miR‐21 on the biological behavior including proliferation and migration of HUVECs. Hence, we used miR‐21 mimics which were transfected directly into HUVECs and we built a cocultured model between HUVECs with EC9706 cells transfected with miR‐21 mimics. The cell proliferation (Figure 4a,b) and cell migration (Figure 4c,d) of HUVECs were increased by 27.65% and 44.41%, respectively compared with the NC mimics group rafter transfection with miR‐21 mimics. When cocultured with EC9706 cells, the HUVECs exhibited significantly increased proliferation by 18.24% (Figure 4e,f) and increased migration by 53.20% (Figure 4g,h) respectively. These results suggest that EC9709‐exo delivered miR‐21 to HUVECs might contribute to promoting tumor growth and metastasis through angiogenesis.

### Exosome‐shuttling miR‐21 promotes angiogenesis by regulating the PTEN/Akt signaling pathway

**FIGURE 5:**
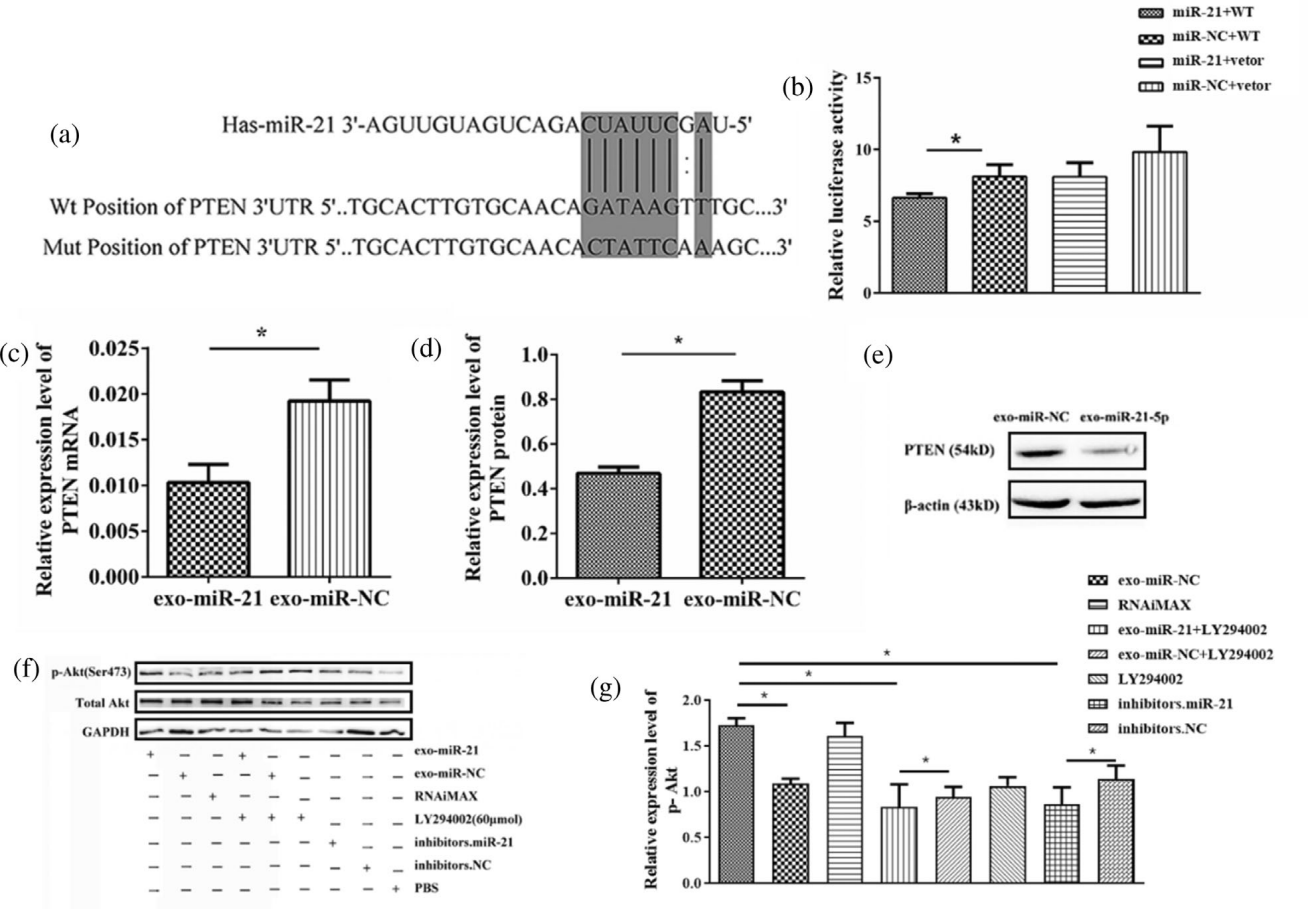
MiR‐21 directly targeted PTEN by binding to its complementary sites in 3′‐UTR. (a) Predicted binding sites of miR‐21 within the 3′‐UTR of PTEN mRNA. (b) Luciferase reporter gene assay showed that cotransfection with miR‐21 mimics significantly repressed the fluorescence ratio of PTEN dependent reporter genes. (c) Real‐time quantitative polymerase chain reaction analysis of PTEN mRNA levels in human umbilical vein endothelial cells (HUVECs) after coculture with EC9706 cells for 24 h. (d) Western blot analysis of PTEN expression in recipient HUVECs after coculture with EC9706 cells for 24 h. (e) The corresponding quantitative analysis of the protein of PTEN. (f) Western blot analysis of Akt and p‐Akt expression in recipient HUVECs treated with LY294002 and miR‐21 inhibitor. (g) The corresponding quantitative analysis of the protein of p‐Akt. *p < 0.05.

We preliminarily confirmed that miR‐21 exhibits a highly conserved ability to directly target the 3′‐UTR of PTEN mRNA across different species based on the bioinformatic analysis (Figure 5a). In addition, a luciferase assay showed that the relative luciferase activity was clearly inhibited when miR‐21 mimics were cotransfected with luciferase reporters and the interaction was lost in the NC group (Figure 5b). RT‐qPCR and western blot analysis were used to detect the PTEN mRNA and protein level in HUVECs after 24 h coculture. PTEN mRNA level as well as the protein level in the exosome‐shuttling miR‐21 group was significantly lower (0.54‐fold) than the NC group (Figure 5c–e), while the miR‐21 inhibitor transfection increased PTEN mRNA level (1.6‐fold) than the miR‐21 group. The results showed that PTEN was a potential target gene of miR‐21. Recently many studies have found that the PTEN/PI3K/Akt signaling pathway is a target of cancer.^28^ Further, we also detected the downstream gene of PTEN, phospho‐Akt (p‐Akt). Compared with the miR‐NC groups, p‐Akt significantly increased about 40.00% (Figure 5f) in HUVECs after coculture with EC9706 cells. However, after being treated with the Akt inhibitor (LY294002, 60 μmol) and miR‐21 inhibitor, the levels of p‐Akt were reduced by 51.68% and 49.93%, respectively. These results indicated that EC9706‐Exo delivered miR‐21 could directly target PTEN/Akt signaling pathway in HUVECs.

## DISCUSSION

In recent years, a growing number of studies have revealed that ESCC is more complicated than previously thought since highly heterogeneity of TME. The TME compositions, including cancer cells, cancer associated fibroblast, endothelial cell and various immune cells, are recognized to regulate the hallmarks of cancers in tumor proliferation, angiogenesis, invasion, and metastasis, as well as chemotherapeutic resistance.^29^, ^30^ Therefore, deciphering the complexity of ESCC microenvironment is fundamental for clarifying the pathogenetic mechanisms and creating effective and precision treatment. With the advancement in research, established the high‐throughput single‐cell RNA sequencing (scRNA‐seq) has been used to dissect the tumor heterogeneity and decipher the interaction between cancer cells and their microenvironment components in head and neck cancer, liver cancer and colorectal tumors.^31^, ^32^, ^33^ Recently, based on scRNA‐seq data, Chen et al. revealed ANXA1/FPR2 signaling as an important crosstalk mechanism between epithelial cells and fibroblasts in promoting ESCC and provided a novel mechanism for the activation of CAFs in the ESCC microenvironment.^34^ Notably, emerging evidences have shown that angiogenesis plays an important role in tumor growth and metastasis especially for most solid tumors, since both expansion of the primary tumor and metastasis to distant organs depend largely on the formation of new blood vessels which provide increased availability of oxygen and nutrients to the tumor.^3^ However, endothelial cells are necessary for angiogenesis, but the endothelial cell heterogeneity of the TME and the pathways and mechanisms of communication with cancer cells remain unclear.

In this study, we extracted and reclustered the endothelial cells by integrating multiple marker genes in all reported ESCC scRNA‐seq results. Here, we identified four endothelial cells subtypes in cancer tissues, including LEC, CAFsEc, EndMT and AEC. Furthermore, we deciphered the communication between endothelial cell subtypes and cancer cells and found that AEC showed a strong interaction with cancer cells in secreted signaling. However, the exact mode of interaction and mechanism are not yet clear.

Recently, the ability of tumor‐derived exosomes (TEX) to induce angiogenesis via modulating mechanisms responsible for the blood vessel development in various cancers has been well documented. For example, exosomes secreted by glioblastomas could promote angiogenesis by increasing tubule length both in vitro and in vivo.^35^, ^36^ The result is supported in other solid tumors, including colorectal cancer,^11^ or pancreatic carcinoma.^10^ Actually, TEX only serve as a way of delivering genetic information in the TME. A possible explanation is that it is the molecules such as miRNAs and proteins packaged in TEX and released in the TME who really take part in angiogenesis. A growing number of studies have reported the role of miRNAs transported by TEX in angiogenesis. Zeng et al. stated that exosomal miR‐25‐3p derived from colorectal cancer cells significantly promoted vascular permeability and angiogenesis in endothelial cells.^11^ Yang et al. demonstrated that exosomes delivered miR‐130a from gastric cancer cells promoted angiogenesis and tumor growth in vascular cells by targeting c‐MYB both in vivo and in vitro.^37^

MiR‐21 has been widely studied and recognized as an oncogene in promoting the progression of various cancers, such as gastric cancer,^38^ lung cancer^39^ and EC.^40^ The regulation of proliferation, invasion, and tumor angiogenesis attributed to miR‐21 has been studied in different cancers.^41^, ^42^ In gastric cancer miR‐21 promoted peritoneal metastasis via mesothelial‐to‐mesenchymal transition, and miR‐21 could also promote gastric tumorigenesis by modulated prostaglandin signaling.^38^, ^43^ In bladder cancer, miR‐21 inhibits autophagy and promotes malignant development of bladder cancer.^44^ MiR‐21 promotes angiogenesis and malignant progression but the mechanism of exosomal miR‐21 via proangiogenic activity in ESCC has not yet been clearly elucidated.^45^ Multiple studies have shown that miR‐21 contained within exosomes play an important role in the TME.^46^ Exosomal miR‐21 participates in the crosstalk between tumor and endothelial cells and induces tumor angiogenesis. Lung cancer cells shuttled exosomal miR‐21 induce vascular VEGF production and angiogenesis.^47^ Our previous studies revealed that miR‐21 was highly expressed in ESCC cells and their corresponding exosomes, which could promote ESCC migration and invasion.^22^ Moreover, the results from the TCGA database also showed that miR‐21 expression was significantly higher in ESCC tissues. Based on the bioinformatic analysis of the target gene of miR‐21, we proved that ESCC cells derived exosomal miR‐21 could promote angiogenesis in endothelial cells via targeting PTEN. According to the coculture model of EC9706 cells and HUVECs, we found that exosomal miR‐21 transported from EC9706 cells enhanced the migration of HUVECs and tube formation in a simulated TME. Additionally, we identified PTEN as a target of miR‐21 in mediating angiogenesis. PTEN is an inhibitor of the PI3K/Akt signaling pathway.^48^, ^49^ It has been widely reported to inhibit angiogenesis via negative regulation of the PI3K/Akt signaling pathway. Cheng et al. reported that astragaloside IV could promote angiogenesis through activating the PI3K/Akt signaling pathway by inhibiting PTEN.^50^ In addition, we proved that p‐Akt was upregulated in HUVECs after coculture with EC9706 cells, while inhibiting the PI3K/Akt pathway and miR‐21, respectively both led to a sharp decrease in p‐Akt levels, suggesting that EC9706 delivered exo‐miR‐21 might increase the activity of Akt signaling via downregulating PTEN. However, VEGF exhibits higher expression in EC tissue and plays a pivotal role in promoting angiogenesis.^51^ Numerous studies have indicated that its regulation is mediated by the PI3K/Akt signaling pathway.^52^, ^53^ Overall, miR‐21 promotes angiogenesis through activation of the PI3K/Akt signaling pathway and subsequent upregulation of VEGF.

However, there were several limitations in this study. First, it was only performed in vitro; however, it remains uncertain whether comparable outcomes can be replicated in vivo. Second, the research mechanism of angiogenesis in ESCC is not comprehensive enough, and the role of downstream molecules of Akt in HUVECs needs to be explored in the future. Moreover, additional validation is required to determine the clinical significance of exosomal miR‐21 and PTEN expression in the plasma of ESCC patients.

**FIGURE 6:**
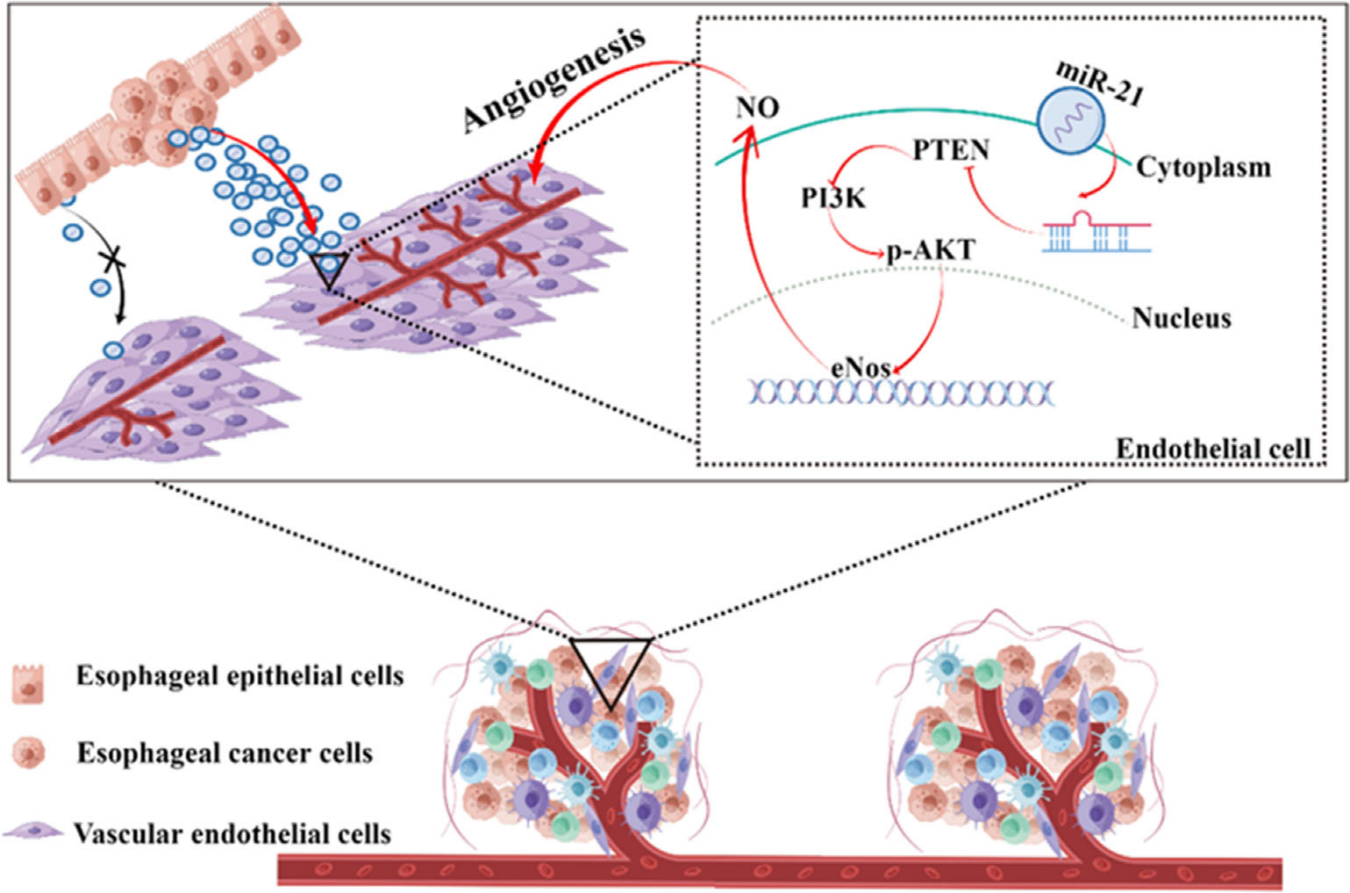
Schematic representation of carcinoma‐derived exosomal miR‐21 promote angiogenesis via regulating the PTEN/Akt signaling pathway in endothelial cells.

In conclusion, we found that endothelial cells in the ESCC microenvironment were highly heterogeneous and can form signal communication with cancer cells. Importantly, miR‐21 via exosome transport could function as an endogenous miRNA in endothelial cells, and exo‐miR‐21 could promote endothelial cell migration and tube formation via targeting PTEN/Akt (Figure 6). Concerning the role of TEX derived miR‐21 in angiogenesis, targeting miR‐21 could be a perspective intervention strategy to prevent carcinogenesis of ESCC in the future.

## AUTHOR CONTRIBUTIONS

Shanbo Zheng: Conceptualization; data curation; writing original draft. Mingjun Sun: Methodology; data collection. Juan Liao: Data analysis and curation. Ran Liu: Funding acquisition. Junjie Lv: Supervision; writing, review and editing.

## FUNDING INFORMATION

This work was supported by the National Natural Science Foundation of China, (grant numbers: 82173479, 81872579) and the Postgraduate Research & Practice Innovation Program of Jiangsu Province, (grant nos.: KYCX22‐0303).

## CONFLICT OF INTEREST STATEMENT

All authors have no conflict of interest.

## Supporting information


**Data S1.** Supporting information.

## Data Availability

The datasets generated during and/or analyzed during the current study are available from the corresponding author on reasonable request.
