# Supplementary material for: Extracellular shuttling miR‐21 contributes to esophageal cancers and human umbilical vein endothelial cell communication in the tumor microenvironment and promotes tumor angiogenesis by targeting phosphatase and tensinhomolog
Source: Thorac Cancer. 2023 Sep 19;14(31):3119–32. doi: 10.1111/1759-7714.15103 (PMC10626251; doi:10.1111/1759-7714.15103)
Supplement: Supplementary file 1 — Data S1. Supporting information. [file TCA-14-3119-s001.docx]

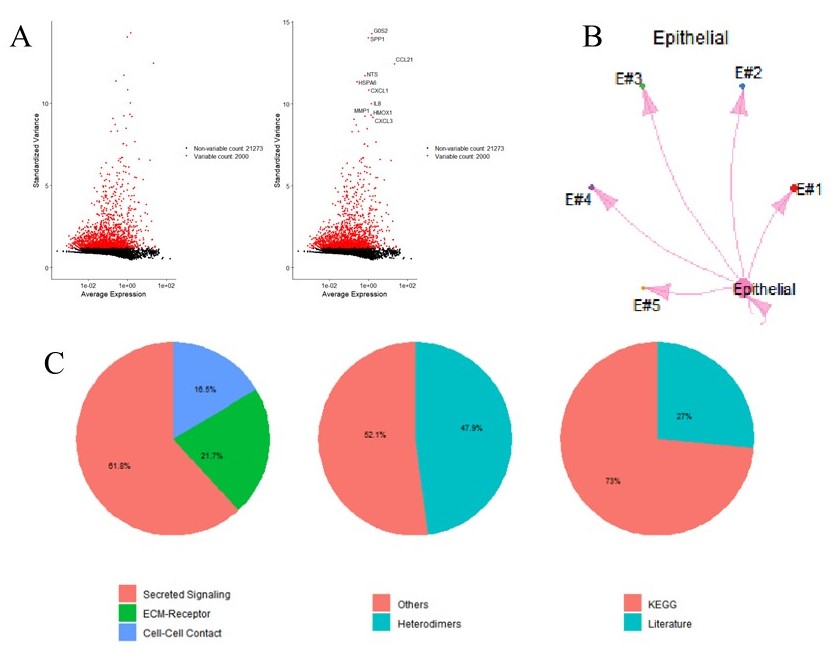


Figure.S1. (A) The variance plot showed 21273 genes in all cells(left), the red dots represent the top 2000 highly variable genes and the black dots represent non-variable genes (right). (B) Detailed view of the ligands expressed by each endothelial cell subtype and cancer cells. (C) The composition of CellPhoneDB database.


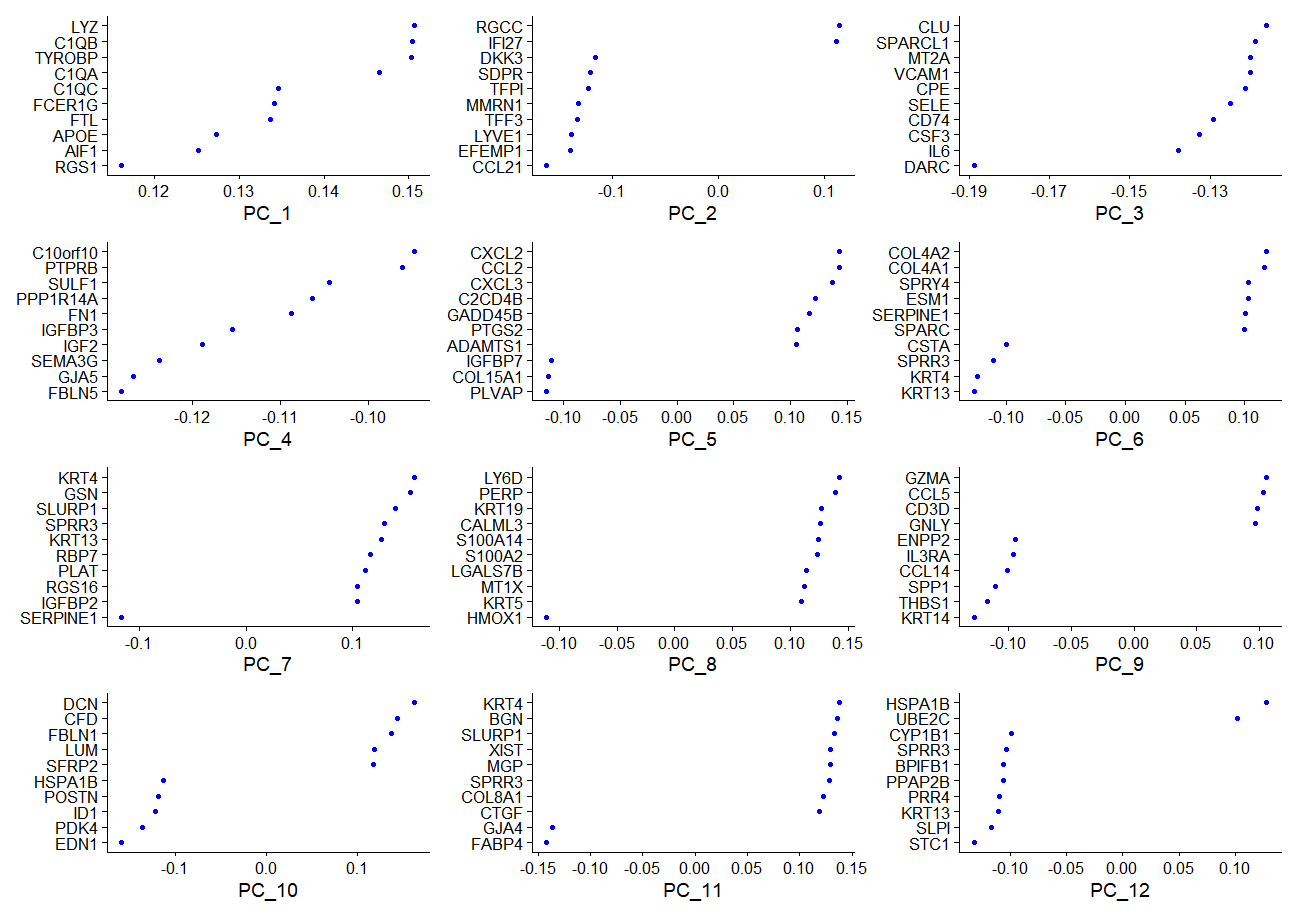


Figure.S2. The top 12 components and the correlated genes in PCA analysis, the top related genes to each principal component.


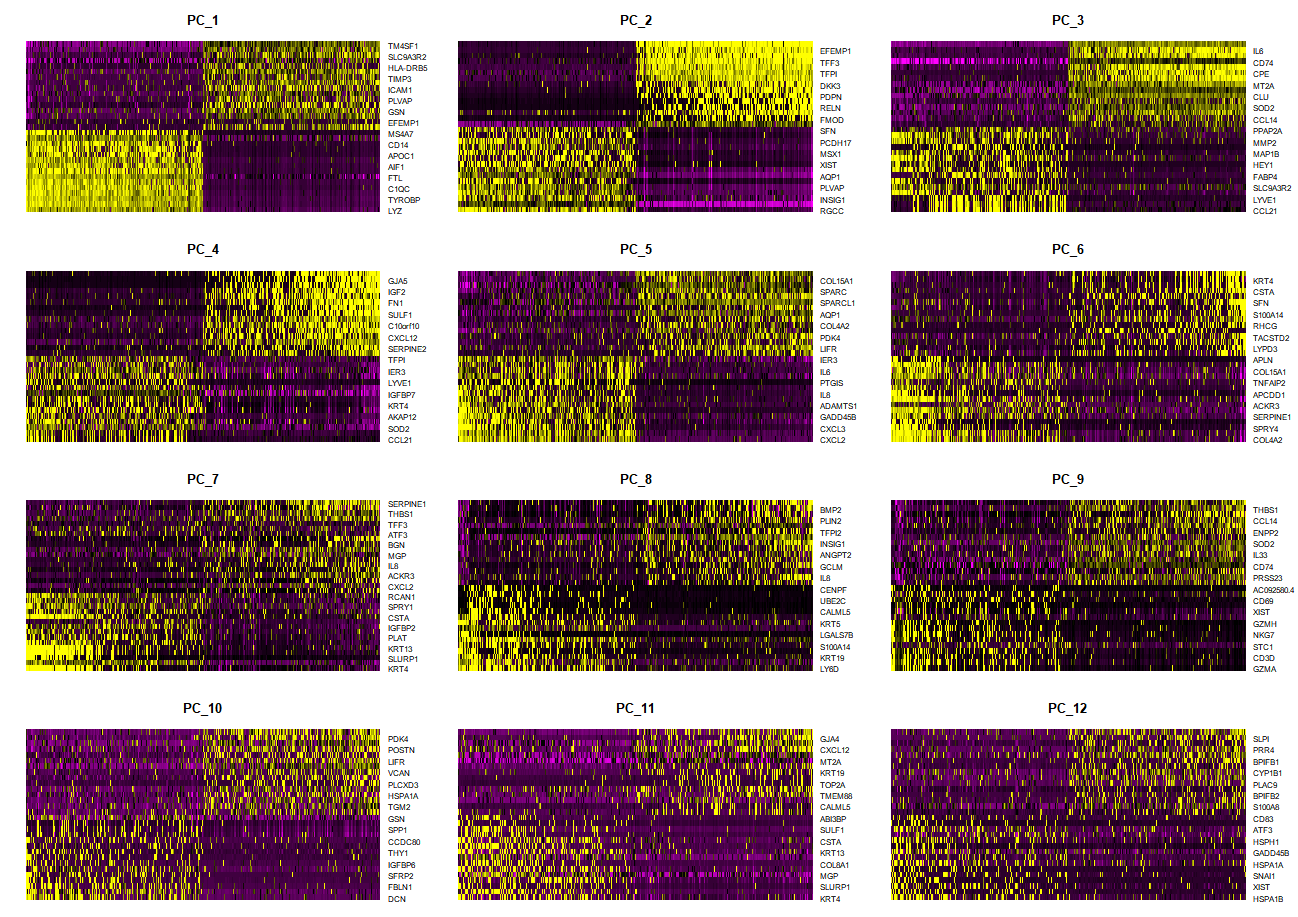


Figure.S3. The heatmap showing the expression level of the top related genes, the colors ranging from purple to yellow represented the expression values from low to high.

Supplement Table 1 The sequences of primer used for RT-qPCR

| Gene | Sequence |
| --- | --- |
| PTEN | Forward: AATGGCTAAGTGAAGATGACAAT |
|  | Reverse: TGCACATATCATTACACCAGTTCGT |
| β-actin | Forward: ATCCGCAAAGACCTGT |
|  | Reverse: GGGTGTAACGCAACTAAG |
